# Protein and antigen profiles of third-stage larvae of *Gnathostoma spinigerum* assessed with next-generation sequencing transcriptomic information

**DOI:** 10.1038/s41598-022-10826-4

**Published:** 2022-04-28

**Authors:** Kathyleen Nogrado, Tipparat Thiangtrongjit, Poom Adisakwattana, Paron Dekumyoy, Sant Muangnoicharoen, Charin Thawornkuno, Onrapak Reamtong

**Affiliations:** 1 Department of Molecular Tropical Medicine and Genetics, Faculty of Tropical Medicine, Mahidol University, Bangkok, 10400 Thailand; 2 Department of Helminthology, Faculty of Tropical Medicine, Mahidol University, Bangkok, 10400 Thailand; 3 Department of Clinical Tropical Medicine, Faculty of Tropical Medicine, Mahidol University, Bangkok, 10400 Thailand

**Keywords:** Biomarkers, Proteomics

## Abstract

Gnathostomiasis is a food-borne zoonotic disease that can affect humans who eat improperly cooked meat containg infective third-stage larvae. Definitive diagnosis is through larval recovery. However, this is an invasive technique and is impractical if the larvae have encysted in inaccessible areas of the body. Antigen or antibody detection might be more interesting techniques for diagnosis. Proteomic could elucidate diagnostic markers and improve our understanding of parasite biology. However, proteomic studies on *Gnathostoma spinigerum* are hampered by the lack of a comprehensive database for protein identification. This study aimed to explore the protein and antigen profiles of advanced third-stage *G. spinigerum* larvae (aL3Gs) using interrogation of mass spectrometry data and an in-house transcriptomic database for protein identification. Immunoproteomic analysis found 74 proteins in 24-kDa SDS-PAGE bands, which is size-specific for the immunodiagnosis of gnathostomiasis. Moreover, 13 proteins were found in 2-DE 24-kDa bands. The data suggest that collagenase 3, cathepsin B, glutathione S-transferase 1, cuticle collagen 14, major antigen, zinc metalloproteinase nas-4, major egg antigen, peroxiredoxin, and superoxide dismutase [Cu–Zn] may be good candidates for novel human gnathostomiasis diagnostic assays. These findings improve our understanding of the parasite’s biology and provide additional potential targets for novel therapeutics, diagnostics, and vaccines.

## Introduction

Human gnathostomiasis is most commonly caused by *Gnathostoma spinigerum*. Infection occurs when humans ingest the infective third-stage larvae through consumption of raw or undercooked meat from second intermediate or paratenic hosts^1^. Gnathostomiasis is an emerging infectious disease with increasing reports of infection, particularly in travelers returning from endemic areas or from importation of infected fresh produce like eels^2^.

Definitive diagnosis of *G. spinigerum* is through identification of the nematode isolated from skin lesions or histopathological sections but this is invasive and is difficult when internal organs are affected. A presumptive diagnosis can be made in patients with eosinophilia coupled with a reported history of eating raw or undercooked fish or meat^3,4^. Another reliable diagnostic method for gnathostomiasis is immunoblotting to detect the 24-kDa crude worm antigen (CWA) from advanced third-stage *G. spinigerum* larvae (aL3Gs)^5^. This *G. spinigerum*-specific antigen has been found in gnathostomiasis and presumptive gnathostomiasis patients, but not in healthy individuals or patients infected with other parasites^6^.

Typically, aL3Gs are harvested from eel livers. The process of CWA production is complex and complicated by the seasonal prevalence of the aL3Gs in eels^7,8^. With advances in proteomics and mass spectrometry, the 24-kDa antigen has been further characterized and found to have high homology to peptide sequence regions of cyclophilin, actin, matrix metalloproteinase-like protein, and intermediate filament protein B. Related studies have shown the applicability of some of these peptides as antigens^9,10^. However, no commercial diagnostic tests are currently available for gnathostomiasis.

For many years, there was no effective treatment for gnathostomiasis and currently the only successful treatment option is surgical excision of the larvae. Various medications, including thiabendazole, praziquantel, metronidazole, diethylcarbamazine, and quinine, have been explored in animal models and humans without success^11^. Proteomic technology has provided crucial information about cellular and molecular processes, leading to an improved understanding of the biology of many different parasites. Aside from identifying antigens for diagnostic purposes, proteomic studies have made invaluable contributions in drug target identification and understanding host-parasite relationships. This work has culminated in the identification of possible drug and vaccine targets for a range of parasites. In contrast, recent proteomic analyses on *G. spinigerum* aL3Gs have only focused on identifying immunoreactive proteins^10,12^. To date, immunoproteomics is the only proteomic analysis that has been used to study *G. spinigerum.* Other proteomic studies have been limited by the lack of a database for *G. spinigerum* protein identification. Therefore, protein identification has mostly relied on using nucleotide sequences from other nematode species. Recently, next-generation sequencing (NGS) was performed to provide a transcriptomic dataset from *G. spinigerum* aL3Gs^4^. This *G. spinigerum* database was applied in this study to facilitate proteomic and immunoproteomic identification of aL3G proteins and antigens. The findings of this study should improve current knowledge of the aL3G proteome and may help to elucidate the molecular functions and biological processes occurring in the parasite to ultimately determine novel drug targets. In addition, these results have identified immunogenic proteins which could be useful for the improvement of vaccines and diagnostic assays for gnathostomiasis.

## Results

### Protein profile of *G. spinigerum* third-stage larvae

**Figure 1:**
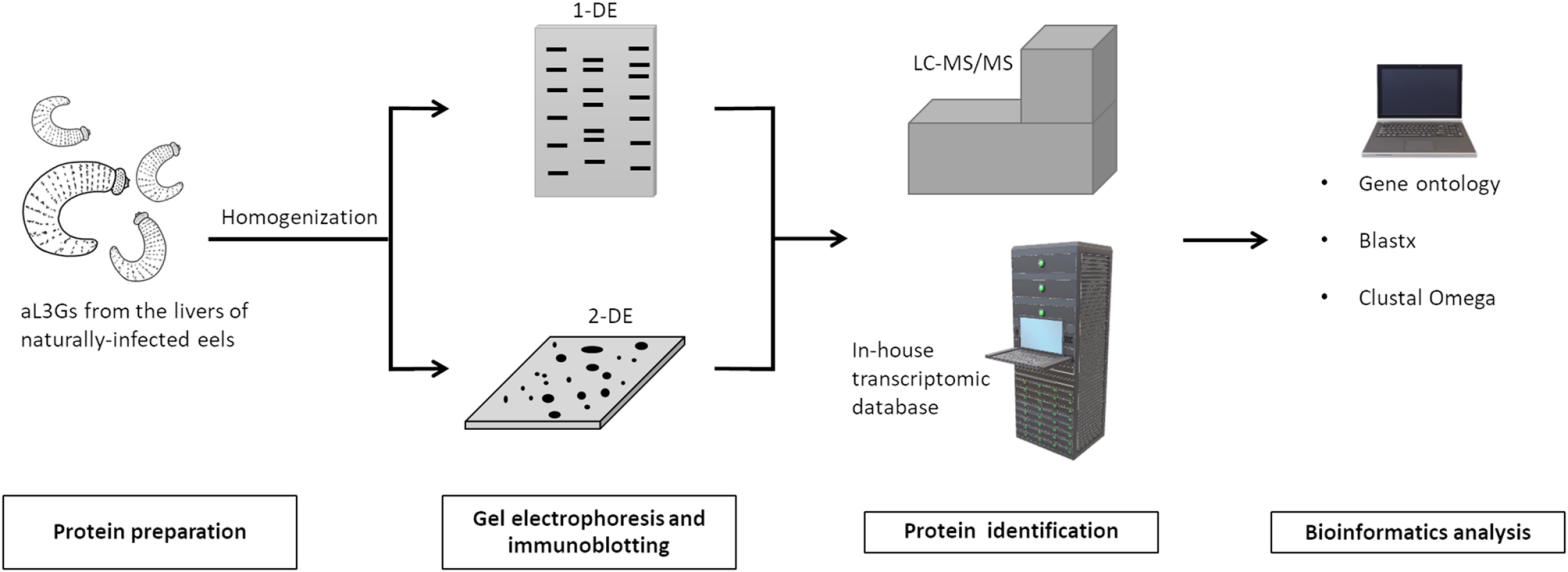
A diagrammatic flowchart summarizing the methods performed in the study.

**Figure 2:**
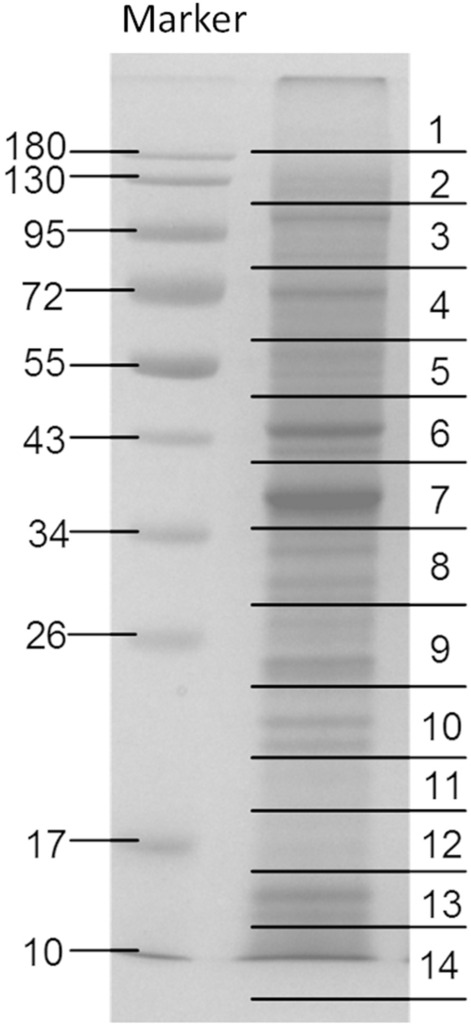
The Coomassie blue-stained gel of aL3Gs proteins. The proteins were separated using one-dimensional gel electrophoresis.

In this research, protein and antigen profiles of aL3G were explored (Fig. 1). Proteins from aL3Gs were separated by 12% gel electrophoresis. A Coomassie blue-stained gel is shown in Fig. 2. A total of 14 gel pieces were cut and in-gel digestion and mass spectrometry analysis were performed. Proteins were identified by searching against the in-house *G. spinigerum* transcriptomic database^4^. With a 95% confidence interval cut-off, 687 proteins were identified (Supplementary Dataset 1). These *G. spinigerum* proteins were semi-quantified based on the exponentially modified protein abundance index (emPAI). The 20 most abundant aL3G proteins are shown in Table 1. The highly abundant proteins had an emPAI value that ranged from 0.99 to 289.02 and molecular weight (MW) that ranged from 7.5 to 250.9 kDa. Among the proteins identified, actin-2 expression increased by 43-fold compared with actin-5c, the second most highly expressed protein. Similarly, other structure-related proteins such as myophilin and actin-1 were also highly expressed in aL3Gs. In addition, several proteases such as zinc metalloprotease nas-14 and matrix metalloproteinase-like protein were also abundant in aL3Gs. Metabolic enzymes, including glyceraldehyde-3-phosphate dehydrogenase and nucleoside diphosphate kinase, were also highly expressed. Furthermore, enzymes responsible for protein folding, such as peptidyl-prolyl-cis–trans-isomerase 3, were also present at high levels in aL3Gs.

**Table 1:** List of the top-20 most abundant proteins in aL3Gs determined based on emPAI values.

No	Accession	Protein description	Score	M.W	No. of peptide	%coverage	emPAI
1	CL1711.Contig5_GsL3-Total-RNA	Actin-2	696.5	7582	9	87.1	289.02
2	Unigene19421_GsL3-Total-RNA	Actin-5C	719	10,846	10	71.6	6.7
3	Unigene18798_GsL3-Total-RNA	Myoglobin	648	24,935	13	48.2	5.48
4	CL1262.Contig3_GsL3-Total-RNA	Ancylostoma secreted protein	643	31,485	11	54.1	2.6
5	CL7245.Contig1_GsL3-Total-RNA	Myophilin	374	25,023	7	30.2	2.33
6	Unigene9624_GsL3-Total-RNA	Glyceraldehyde-3-phosphate dehydrogenase	849	44,630	17	47.5	2.13
7	CL3958.Contig3_GsL3-Total-RNA	Small heat shock protein OV25-1	307	30,262	12	50.2	2.03
8	Unigene14002_GsL3-Total-RNA	Peptidyl-prolyl cis–trans isomerase 3	228	31,937	8	33.2	1.58
9	CL3958.Contig1_GsL3-Total-RNA	Small heat shock protein OV25-1	263	30,805	9	36.6	1.39
10	CL7161.Contig1_GsL3-Total-RNA	Zinc metalloproteinase nas-14	253	15,103	6	43.3	1.37
11	CL1711.Contig2_GsL3-Total-RNA	Actin, cytoplasmic 1	731	51,490	14	26.8	1.36
12	Unigene6082_GsL3-Total-RNA	Nucleoside diphosphate kinase	358	26,046	8	30.9	1.16
13	CL3580.Contig2_GsL3-Total-RNA	Collagenase 3	236	31,095	10	32.6	1.13
14	Unigene20056_GsL3-Total-RNA	Zinc metalloproteinase nas-14	209	17,429	9	67.1	1.12
15	Unigene18966_GsL3-Total-RNA	Small heat shock protein OV25-2	91	32,250	12	47.6	1.07
16	Unigene9664_GsL3-Total-RNA	Transthyretin-like protein 16	158	18,249	4	25.9	1.06
17	Unigene18725_GsL3-Total-RNA	Calponin homolog OV9M	643	72,439	23	38.5	1.03
18	Unigene21925_GsL3-Total-RNA	Actin-1	897	73,407	23	32.1	1.01
19	Unigene16420_GsL3-Total-RNA	Extracellular globin	360	49,428	16	41.9	0.99
20	Unigene21322_GsL3-Total-RNA	Major antigen	4688	250,951	80	34	0.99

**Figure 3:**
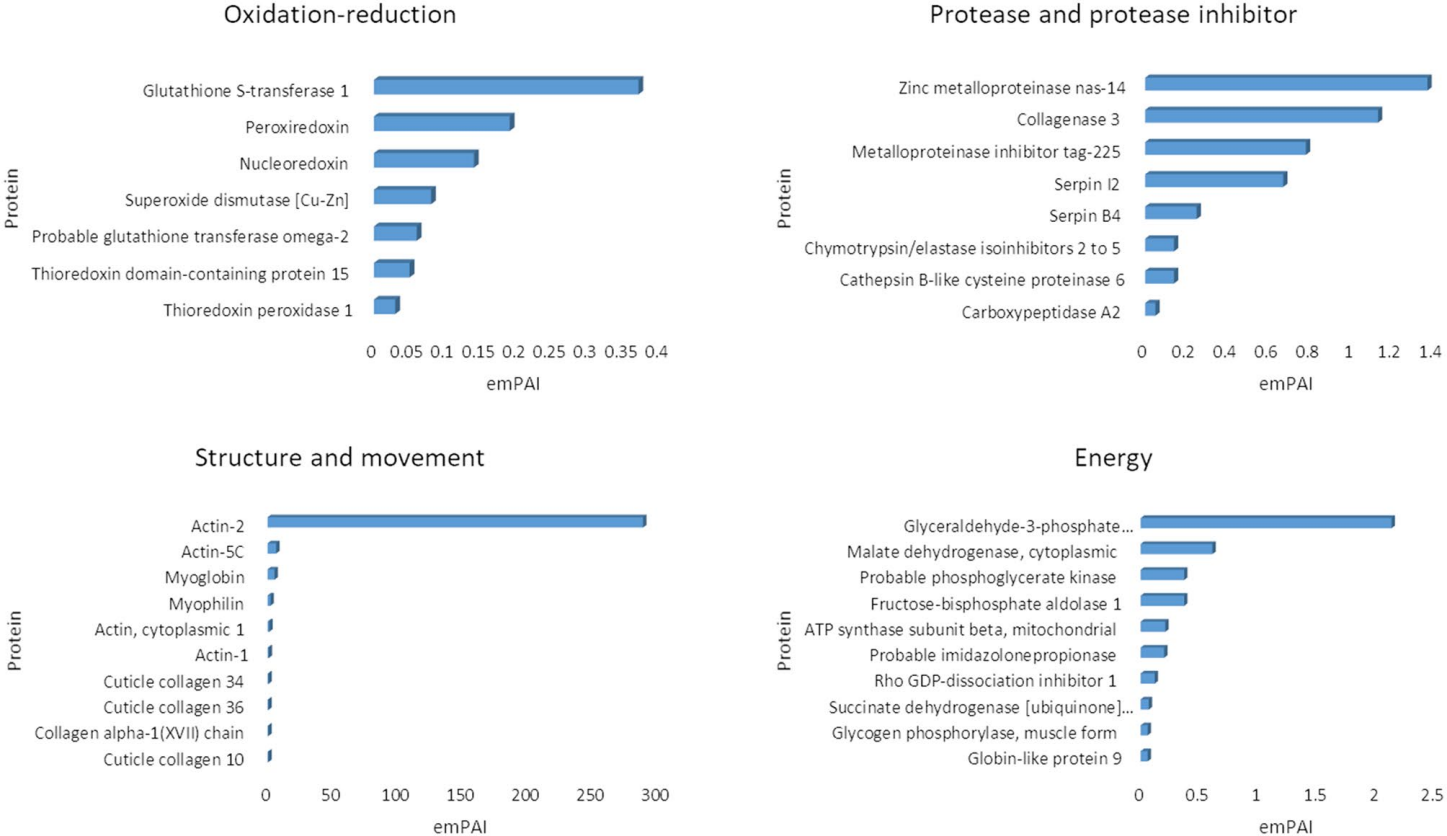
The top-10 most abundant proteins classified by molecular function relating to oxidation–reduction (redox), protease-protease inhibitor activity, structure-movenent, and energy metabolism. Their abundance was estimated semi-quantitatively by emPAI. In each category, the x-axis indicates the emPAI value and the y-axis specifies the protein identification.

To gain more understanding into the biological processes occurring in aL3Gs, gene ontology (GO) was used to classify all identified *G. spinigerum* proteins (Supplementary Table 1). A total of 123 protein classes were found in aL3Gs. The top 20 protein classes are listed in Table 2. Proteins with unknown GO accounted for approximately 50% of the total identified *G. spinigerum* proteins. The major annotated protein classes observed in aL3Gs included embryo development ending in birth or egg hatching (GO:0009792), determination of adult lifespan (GO:0008340), and oviposition (GO:0018991). Other protein classes were involved in biological processes related to energy, movement, morphological development, and enzymes for protein structure conformational changes. In addition to GO classification by biological process terms, proteins essential for parasite survival and host immunity evasion were also considered. Proteins relating to oxidation–reduction, proteinase-protease inhibitors, structure-movement, and energy were identified in the aL3G protein profile. The top 10 most abundant *G. spinigerum* proteins involved in these four categories are shown in Fig. 3. Glutathione S-transferase (emPAI of 0.37), peroxiredoxin (emPAI of 0.19), and nucleoredoxin (emPAI of 0.14) were the major antioxidant proteins expressed in aL3Gs. The most prevalent proteases were zinc metalloproteinase nas-14 (emPAI of 1.37), matrix metalloproteinase-like protein (emPAI of 1.13), and cathepsin B-like cysteine proteinase 6 (emPAI of 0.14), while the most abundant protease inhibitors were metalloproteinase inhibitor tag-225 (emPAI of 0.78), serpin I2 (emPAI 0.67), and serpin B4 (emPAI of 0.25). Meanwhile, actin-2 (emPAI of 289.02), actin-5C (emPAI of 6.7), and myoglobin (emPAI of 5.48) were the most highly expressed proteins in the structure-movement group. Additionally, glyceraldehyde-3-phosphate dehydrogenase (emPAI of 2.13), malate dehydrogenase, cytoplasmic (emPAI of 0.61), and phosphoglycerate kinase (emPAI of 0.37) were the main energy-related proteins expressed in aL3Gs.

**Table 2:** List of top-20 proteins classified by Gene Ontology (GO) according to the biological processes involved in aL3Gs.

GO-Biological Process	No. of proteins
Unknown	401
GO:0,009,792//embryo development ending in birth or egg hatching	33
GO:0,008,340//determination of adult lifespan	24
GO:0,018,991//oviposition	11
GO:0,006,096//glycolytic process	7
GO:0,008,152//metabolic process	7
GO:0,019,915//lipid storage	7
GO:0,055,085//transmembrane transport	6
GO:0,000,003//reproduction	5
GO:0,010,171//body morphogenesis	5
GO:0,040,011//locomotion	5
GO:0,040,035//hermaphrodite genitalia development	5
GO:0,006,094//gluconeogenesis	4
GO:0,006,457//protein folding	4
GO:0,009,987//cellular process;GO:0,044,238	4
GO:0,016,310//phosphorylation	4
GO:0,030,968//endoplasmic reticulum unfolded protein response	4
GO:0,044,763;GO:0,050,794//regulation of cellular process	4
GO:0,048,856//anatomical structure development	4
GO:0,071,688//striated muscle myosin thick filament assembly	4

### Comparison of aL3G and human protein sequences

To identify potential *G. spinigerum* drug target candidates, the nucleotide sequences of the 10 most abundant proteins relating to oxidation–reduction, proteinase-protease inhibitors, and structure-movement were retrieved from the aL3G transcriptome. The blastx algorithm was used to search the lowest E-value for human proteins in the GenBank database compared with *G. spinigerum* sequences. The percent homology between *G. spinigerum* and human sequences is shown in Table 3.

**Table 3:** List of Abundant proteins of aL3Gs classified by Gene Ontology (GO) into four groups under the molecular function category that are aligned with human proteins to identify unique proteins of aL3Gs.

Protein	Accession no. of human proteins	%identity	E-value	Query cover
**Oxidation–reduction**				
Thioredoxin peroxidase 1	NP_006784.1	62.23%	3.00E-79	17%
Thioredoxin domain-containing protein 15	NP_001337664.1	30.34%	2.00E-16	22%
Probable glutathione transferase omega-2	4YQM_A	34.85%	9.00E-36	42%
Superoxide dismutase [Cu–Zn]	3GTV_A	58.39%	1.00E-51	37%
Nucleoredoxin	NP_001155097.1	36.36%	8.00E-28	61%
Peroxiredoxin	NP_005800.3	74.61%	8.00E-102	34%
Glutathione S-transferase 1	4EDY_A	40.00%	6.00E-08	68%
**Protease-protease inhibitor**				
Carboxypeptidase A2	AAH14571.1	37.98%	8.00E-88	63%
Cathepsin B-like cysteine proteinase 6	NP_001304166.1	38.36%	6.00E-29	69%
Chymotrypsin/elastase isoinhibitors 2 to 5*	–	–	–	–
Serpin B4*	–	–	–	–
Serpin I2*	–	–	–	–
Metalloproteinase inhibitor tag-225*	–	–	–	–
Collagenase 3	BAD96700.1	36.99%	6.00E-29	61%
Zinc metalloproteinase nas-14*	–	–	–	–
**Structure-movement**				
Actin-2	BAG51757.1	98.55%	9.00E-45	98%
Actin-5C	BAG62762.1	97.40%	5.00E-50	75%
Myoglobin*	–	–	–	–
Myophilin	EAW52759.1	38.52%	4.00E-24	55%
Actin, cytoplasmic 1	AAH10417.2	98.16%	9.00E-115	34%
Actin-1	NP_001186883.1	92.51%	0.00E + 00	57%
Cuticle collagen 34*	–	–	–	–
Cuticle collagen 36*	–	–	–	–
Collagen alpha–1(XVII) chain*	–	–	–	–
Cuticle collagen 10*	–	–	–	–
**Energy metabolism**				
Glycogen phosphorylase, muscle form	1XOI_A	67.45%	0.00E + 00	74%
Succinate dehydrogenase [ubiquinone] flavoprotein subunit, mitochondrial	XP_016865174.1	80.62%	0.00E + 00	85%
Rho GDP-dissociation inhibitor 1	BAG35268.1	48.08%	9.00E-52	34%
Probable imidazolonepropionase	NP_689648.2	49.42%	3.00E-136	81%
ATP synthase subunit beta, mitochondrial	BAA00016.1	84.43%	0.00E + 00	73%
Fructose-bisphosphate aldolase 1	NP_001121089.1	67.88%	1.00E-171	72%
Probable phosphoglycerate kinase	4AXX_A	73.32%	0.00E + 00	81%
Malate dehydrogenase, cytoplasmic	NP_005908.1	62.69%	5.00E-142	84%
Glyceraldehyde-3-phosphate dehydrogenase	6M61_O	73.53%	3.00E-168	81%
Globin-like protein 9*	–	–	–	–

According to the alignment results, 10 *G. spinigerum* proteins including thioredoxin domain-containing protein 15, glutathione transferase omega-2, nucleoredoxin, glutathione S-transferase 1, carboxypeptidase A2, cathepsin B-like cysteinase 6, collagenase 3, myophilin, rho GDP-dissociation inhibitor 1, and imidazolonepropionase showed less than 50% homology to human sequences. Furthermore, no homology to human proteins was found in the following *G. spinigerum* proteins: chymotrypsin/elastase isoinhibitors 2 and 5; serpin B4; serpin I2; metalloproteinase inhibitor tag-225; zinc metalloproteinase nas-14; myoglobin; cuticle collagen 34; cuticle collagen 36; collagen alpha-1 (XVII) chain; and globin-like protein 9. Therefore, these *G. spinigerum* proteins might be potential candidates for drug development.

### Protein identification in 24-kDa gel bands

**Figure 4:**
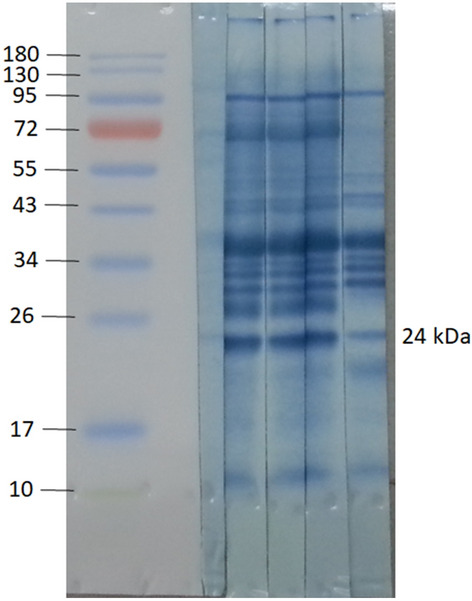
Western blot analyses of aL3Gs with the molecular weight (MW) markers indicated in kDa on the left. Each membrane strip (1–5) is incubated with sera from 5 different individuals diagnosed with gnathostomiasis. The 24-kDa gel regions were excised, and protein components explored against transcriptomic database individually.

Because identification of the 24-kDa aL3G crude worm antigen is an accepted diagnostic assay for gnathostomiasis, the aL3G proteins were separated by 12% gel electrophoresis then electro-transferred onto a membrane. Western blot analysis was performed using the sera of five individual patients with a confirmed diagnosis of gnathostomiasis as primary antibodies (Fig. 4). The aL3G proteins were transferred onto a membrane. The membrane was sliced into five 3 mm-strips. Each membrane strip (1–5) was individually incubated with sera from 5 different individuals diagnosed with gnathostomiasis As expected, the patient sera reacted with gel bands of approximately 24 kDa, a result reported to have high sensitivity and specificity for *G. spinigerum* diagnosis. Gel band numbers 9, 10, and 11 were excised for protein identification by mass spectrometry. The mass spectrometry data from three of the gel pieces were separately searched against NCBI and transcriptomic databases to identify their protein components (Table 4). Using the NCBI database, proteins from gel section numbers 9, 10, and 11 were identified as matrix metalloproteinase-like protein, unknown, and cyclophilin, respectively. Using the *G. spinigerum* transcriptomic database, matrix metalloproteinase-like protein and cyclophilin were identified from gels 9 and 11, respectively. Furthermore, 37, 26, and 23 proteins were also identified on gels 9, 10, and 11 using our database (Table 4). These data indicate the presence of other intriguing candidates, including antioxidative enzymes, cuticle collagens, and major antigens, which warrant further investigation as potential diagnostic targets.

**Table 4:** The proteins identified from gel numbers 9, 10, and 11 using NCBI and transcriptomic data.

Gel	NCBI	Protein description	Transcriptome	Protein description
9	AAF82802.1	Matrix metalloproteinase-like protein [*Gnathostoma spinigerum*]	CL3580.Contig2_GsL3-Total-RNA	Collagenase 3
			Unigene18966_GsL3-Total-RNA	Small heat shock protein OV25-2
			CL1711.Contig1_GsL3-Total-RNA	Actin-2
			Unigene9422_GsL3-Total-RNA	Triosephosphate isomerase
			Unigene21055_GsL3-Total-RNA	Glutamate dehydrogenase, mitochondrial
			CL6152.Contig2_GsL3-Total-RNA	Glutathione S-transferase 1
			CL3958.Contig1_GsL3-Total-RNA	Small heat shock protein OV25-1
			Unigene18018_GsL3-Total-RNA	Succinate dehydrogenase [ubiquinone] iron-sulfur subunit, mitochondrial
			Unigene16324_GsL3-Total-RNA	Cuticle collagen 14
			Unigene6075_GsL3-Total-RNA	Protein lethal(2)essential for life
			Unigene17956_GsL3-Total-RNA	Adenylate kinase isoenzyme 1
			CL7292.Contig2_GsL3-Total-RNA	Stromelysin-2
			Unigene9624_GsL3-Total-RNA	Glyceraldehyde-3-phosphate dehydrogenase
			Unigene6072_GsL3-Total-RNA	Twitchin
			CL3283.Contig1_GsL3-Total-RNA	Loricrin
			Unigene21925_GsL3-Total-RNA	Actin-1
			CL4911.Contig1_GsL3-Total-RNA	Myb-like protein D
			Unigene21322_GsL3-Total-RNA	Major antigen
			Unigene21850_GsL3-Total-RNA	Probable maleylacetoacetate isomerase
			Unigene21057_GsL3-Total-RNA	Zinc metalloproteinase nas-4
			Unigene1222_GsL3-Total-RNA	Phosphoenolpyruvate carboxykinase [GTP]
			CL2394.Contig1_GsL3-Total-RNA	Probable glutathione transferase omega-2
			CL845.Contig1_GsL3-Total-RNA	Uncharacterized protein ZK643.6
			CL437.Contig1_GsL3-Total-RNA	Disorganized muscle protein 1
			Unigene22528_GsL3-Total-RNA	Elongation factor Ts, mitochondrial
			Unigene18459_GsL3-Total-RNA	Phosphoenolpyruvate carboxykinase [GTP]
			Unigene21888_GsL3-Total-RNA	Propionyl-CoA carboxylase beta chain, mitochondrial
			Unigene22645_GsL3-Total-RNA	E3 ubiquitin-protein ligase pellino homolog 2
			Unigene85_GsL3-Total-RNA	Eukaryotic translation initiation factor 4B
			Unigene24479_GsL3-Total-RNA	Hypoxia up-regulated protein 1
			CL3117.Contig10_GsL3-Total-RNA	Nucleolar protein 10
			Unigene17966_GsL3-Total-RNA	Phosphoenolpyruvate carboxykinase [GTP]
			CL1888.Contig1_GsL3-Total-RNA	Uncharacterized protein ZK688.3
			CL756.Contig10_GsL3-Total-RNA	GRIP1-associated protein 1
			CL607.Contig12_GsL3-Total-RNA	Probable splicing factor, arginine/serine-rich 7
			CL3810.Contig4_GsL3-Total-RNA	Troponin T
			CL3881.Contig1_GsL3-Total-RNA	cathepsin B [EC:3.4.22.1]
			CL2383.Contig1_GsL3-Total-RNA	Transformation/transcription domain-associated protein
10	unknown	Unknown	CL3958.Contig1_GsL3-Total-RNA	Small heat shock protein OV25-1
			CL6067.Contig1_GsL3-Total-RNA	Small heat shock protein OV25-2
			Unigene19421_GsL3-Total-RNA	Actin-5C
			Unigene17198_GsL3-Total-RNA	Major egg antigen
			CL187.Contig1_GsL3-Total-RNA	Phosphatidylethanolamine-binding protein homolog F40A3.3
			Unigene18966_GsL3-Total-RNA	Small heat shock protein OV25-2
			CL2984.Contig1_GsL3-Total-RNA	Beta-ureidopropionase
			CL221.Contig3_GsL3-Total-RNA	Peroxiredoxin
			CL1711.Contig1_GsL3-Total-RNA	Actin-2
			CL5074.Contig2_GsL3-Total-RNA	Elongation of very long-chain fatty acids protein 5
			Unigene20106_GsL3-Total-RNA	Methyltransferase-like protein 17, mitochondrial
			CL6024.Contig2_GsL3-Total-RNA	Ferric-chelate reductase 1
			Unigene1383_GsL3-Total-RNA	E3 ubiquitin-protein ligase MYLIP-B
			CL2656.Contig4_GsL3-Total-RNA	CDKN2AIP N-terminal-like protein
			CL5237.Contig1_GsL3-Total-RNA	DNA polymerase delta subunit 2
			Unigene19315_GsL3-Total-RNA	Rho GDP-dissociation inhibitor 1
			Unigene21055_GsL3-Total-RNA	Glutamate dehydrogenase, mitochondrial
			Unigene2602_GsL3-Total-RNA	Carboxypeptidase A2
			CL5122.Contig1_GsL3-Total-RNA	Barrier-to-autointegration factor 1
			Unigene21322_GsL3-Total-RNA	Major antigen
			Unigene25087_GsL3-Total-RNA	Bromodomain and WD repeat-containing protein 3
			CL2434.Contig1_GsL3-Total-RNA	Thioredoxin peroxidase 1
			CL68.Contig11_GsL3-Total-RNA	Coiled-coil domain-containing protein 18
			CL7147.Contig1_GsL3-Total-RNA	Lipoma-preferred partner homolog
			CL498.Contig10_GsL3-Total-RNA	Stromal interaction molecule 1
			CL1701.Contig10_GsL3-Total-RNA	DmX-like protein 2
11	ACX47902.1	Cyclophilin [*Gnathostoma spinigerum*]	Unigene14002_GsL3-Total-RNA	Peptidyl-prolyl cis–trans isomerase 3 (cyclophilin)
			Unigene9664_GsL3-Total-RNA	Transthyretin-like protein 16
			Unigene16365_GsL3-Total-RNA	OV-16 antigen
			Unigene6082_GsL3-Total-RNA	Nucleoside diphosphate kinase
			CL1711.Contig1_GsL3-Total-RNA	Actin-2
			CL7161.Contig1_GsL3-Total-RNA	Zinc metalloproteinase nas-14
			CL6541.Contig1_GsL3-Total-RNA	Transthyretin-like protein 46
			Unigene26369_GsL3-Total-RNA	Putative uncharacterized transposon-derived protein F52C9.6
			CL221.Contig3_GsL3-Total-RNA	Peroxiredoxin
			Unigene14764_GsL3-Total-RNA	60S ribosomal protein L12
			CL3958.Contig1_GsL3-Total-RNA	Small heat shock protein OV25-1
			CL187.Contig1_GsL3-Total-RNA	Phosphatidylethanolamine-binding protein homolog F40A3.3
			Unigene20506_GsL3-Total-RNA	Superoxide dismutase [Cu–Zn]
			Unigene20053_GsL3-Total-RNA	Myosin, essential light chain
			Unigene9624_GsL3-Total-RNA	Glyceraldehyde-3-phosphate dehydrogenase
			Unigene21322_GsL3-Total-RNA	Major antigen
			CL1294.Contig1_GsL3-Total-RNA	Intermediate filament protein B
			CL1614.Contig1_GsL3-Total-RNA	Endoplasmic reticulum-Golgi intermediate compartment protein 3
			CL198.Contig1_GsL3-Total-RNA	Mitochondrial Rho GTPase
			Unigene25389_GsL3-Total-RNA	Beta-lactamase domain-containing protein 2
			CL7147.Contig1_GsL3-Total-RNA	Lipoma-preferred partner homolog
			Unigene22939_GsL3-Total-RNA	DDB1- and CUL4-associated factor 5
			CL756.Contig10_GsL3-Total-RNA	GRIP1-associated protein 1

### 2D-immunoblot aL3G antigen profile

**Figure 5:**
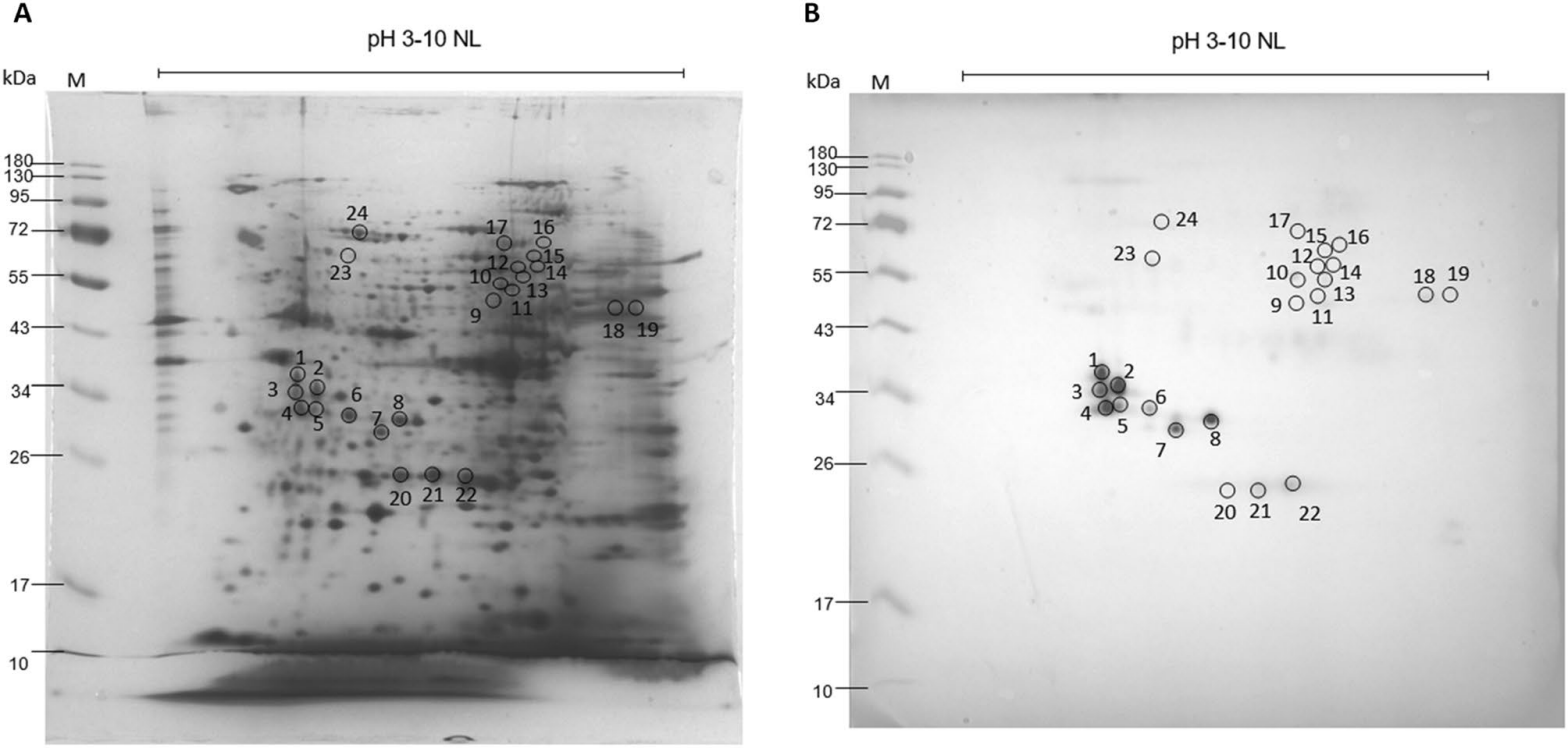
Images of protein from crude extract of aL3Gs subjected to two-dimensional electrophoresis (2-DE). Using IEF, the proteins are separated on a linear pH range of 3–10 in the first dimension and followed by 12% SDS-PAGE for the second dimension. The gel on the left is stained with Coomassie blue (**A**). The right side shows the nitrocellulose membrane with proteins electro-transferred from the gel and then probed with pooled sera from patients diagnosed with gnathostomiasis (**B**). Numbers at the left of each image indicate protein molecular weight (MW) markers.

To explore the aL3G immunome, aL3G proteins were separated by 2-DE and transferred onto a nitrocellulose membrane and later analyzed by western blotting (Fig. 5). Pooled patient sera served as the primary antibody. A total of 24 immunoreactive spots was observed. These spots were identified by comparing mass spectrometry data with the *G. spinigerum* transcriptomic database. A total of 115 proteins were identified as *G. spinigerum* antigens (Table 5). Spots 8, 17, and 22 demonstrated a lot number of protein identification at 21, 17 and 12, respectively. The proteins 32-kDa beta-galactoside-binding lectin, alkylated DNA repair protein alkB homolog 8, and mitogen-activated protein kinase were identified with the highest score in spot 8. Polyphosphoinositide phosphatase, UPF0378 protein, and heat shock factor binding protein 1 were observed with the highest confidence in gel 17. Matrix metalloproteinase-like protein, e3 ubiquitin-protein ligase trim13, and isocitrate isopropylmalate dehydrogenase domain containing protein were found in gel 22.

**Table 5:** List of proteins identified from aL3Gs after 2D-immunoblot analysis to identify proteins of importance related to diagnostic development of gnathostomiasis infection.

Gel	No	Protein description	Score	M.W	No. of sequence	%cov
1	1	Unknown	63	28,771	4	16.2
	2	Unknown	42	54,746	1	1.6
2	1	Unknown	70	28,771	4	19.7
	2	Cytochrome c oxidase assembly protein cox15-like protein	30	64,100	3	7.5
	3	Grip and coiled-coil domain-containing protein 2	30	80,345	2	3.4
	4	f-box wd repeat-containing protein 5	29	21,484	1	5
	5	Hemicentin-1	25	9709	1	8.5
	6	unknown	24	71,901	1	3.1
3	1	unknown	37	28,771	2	7.7
	2	unknown	35	9753	1	9.2
	3	unknown	26	8063	1	21.9
4	1	unknown	130	28,771	4	16.2
	2	Translational activator GCN1	55	293,891	4	2.3
	3	Adenosine monophosphate-protein transferase FICD	52	92,486	3	2.9
	4	Otopetrin-1	48	67,029	2	2.8
5	1	Otopetrin-1	74	131,621	4	4.8
	2	Nucleoside-diphosphatase mig-	55	58,592	2	3.6
	3	Anaphase-promoting complex subunit 5	52	119,152	3	2.7
	4	Trafficking protein particle complex subunit 8	51	154,261	3	2.8
	5	zinc metallopeptidase 2 MEP2	48	74,140	3	5
6	1	Steroidogenic acute regulatory-like protein 1	132	43,117	11	19.7
	2	Ankyrin-2	36	74,803	1	2.4
	3	Methionine synthase reductase	30	90,007	1	1.1
	4	Cytochrome c oxidase assembly protein cox15-like protein	30	63,522	2	3.3
	5	Grip and coiled-coil domain-containing protein 2	29	80,285	2	2.1
	6	Transcription factor Dp-	23	110,765	2	2.6
	7	sn1-specific diacylglycerol lipase beta	19	80,114	2	2.5
7	1	Unknown	78	28,771	2	6.6
	2	Adenosine monophosphate-protein transferase FICD homolog	55	92,486	2	1.8
8	1	32 kDa beta-galactoside-binding lectin	892	43,181	17	34.7
	2	Unknown	52	13,040	1	7.4
	3	Alkylated DNA repair protein alkB homolog 8	48	39,451	2	2.5
	4	Unknown	48	25,280	1	3.5
	5	Mitogen-activated protein kinase 15	48	117,237	1	0.7
	6	Unknown	35	54,746	1	1.6
	7	sh2 domain-containing protein 3c	34	86,816	2	2.3
	8	Cytochrome c oxidase assembly protein cox15-like protein	29	63,522	1	1.4
	9	Protein HID1	29	194,912	3	1.6
	10	Ubiquitin carboxyl-terminal hydrolase 31	29	174,633	1	0.5
	11	Grip and coiled-coil domain-containing protein 2	29	77,955	1	1.2
	12	e3 ubiquitin-protein ligase trim13	27	92,227	1	1.1
	13	putative ubiquitin carboxyl-terminal hydrolase 46	23	83,972	1	1.2
	14	Sestrin-1	23	45,954	1	2.1
	15	Metallophosphoesterase	23	111,504	3	3.2
	16	Ras GTPase-activating protein gap-2	23	195,165	1	0.6
	17	1-acyl-sn-glycerol-3-phosphate acyltransferase alpha	23	77,675	1	1.3
	18	2-oxoglutarate	23	115,162	1	0.9
	19	Magnesium transporter 1	23	108,173	1	0.9
	20	Homeobox protein cut-like ceh-	23	120,530	1	0.9
	21	Putative leucine carboxyl methyltransferase 1	22	69,138	1	1.1
9	1	Fructose-bisphosphate aldolase 1	79	53,610	4	12.6
	2	Unknown	64	151,052	4	2.7
	3	Spectrin beta chain	63	337,895	5	1.9
	4	Proteasome activator complex subunit 4	52	168,147	4	3.5
	5	Nucleoside-diphosphatase mig-23	51	58,592	2	3.6
	6	Uncharacterized protein F54D1.6	51	181,401	3	2.3
	7	Uncharacterized protein DDB_G0271670	50	170,273	4	3.3
10	1	Unknown	33	54,746	1	1.6
	2	Protogenin B (Fragment)	27	239,030	2	1.3
	3	Probable phosphoserine aminotransferase	16	71,802	1	2.8
	4	2-methoxy-6-polyprenyl- -benzoquinol	16	74,095	1	2.6
	5	von Willebrand factor and Proteinase inhibitor I15 domain-containing protein	16	197,901	1	0.9
	6	Unknown	16	27,113	1	7
	7	Decaprenyl-diphosphate synthase subunit 1	16	70,245	1	3
11	1	Fructose-bisphosphate aldolase 1	344	56,589	12	23.4
	2	Fructose-bisphosphate aldolase 1	198	53,610	10	23.5
	3	Unknown	41	54,746	1	1.6
	4	Myosin-2	33	21,125	1	7.1
	5	Transcriptional regulator ATRX	21	100,673	1	1.2
12	1	Neuroserpin	68	274,509	2	0.6
	3	DNA polymerase kappa	35	53,850	1	1.8
	2	Unknown	35	9753	1	9.2
13	1	DNA polymerase kappa	28	53,850	1	1.8
14	-	-	-	-	-	-
15	1	Putative helicase mot1	74	265,846	5	2.5
	2	Protein split ends	60	310,778	5	2.2
	3	Lon protease homolog, mitochondrial	52	277,509	3	1.1
	4	NAD(P) transhydrogenase, mitochondrial	51	128,218	4	5.1
16	1	Unknown	39	54,746	1	1.6
17	1	Unknown	40	30,189	1	3.7
	2	Polyphosphoinositide phosphatase	34	134,822	1	0.7
	3	Unknown	25	33,690	1	4.3
	4	UPF0378 protein	25	295,143	2	1
	5	Heat shock factor binding protein 1	25	44,624	1	4.9
	6	Unknown	22	7327	1	9.1
	7	Nuclear pore complex protein nup160	22	182,675	1	0.7
	8	Unknown	17	51,481	1	2.8
	9	Unknown	17	15,910	1	9.5
	10	Transgelin-2	17	38,743	1	4.2
	11	Unknown	17	27,190	1	4.4
	12	Multiple C2 and transmembrane domain-containing protein 1	17	117,365	2	2.5
	13	Tyrosine-protein phosphatase non-receptor type 13	17	212,990	1	0.7
	14	Reverse transcriptase	17	69,975	1	2.2
	15	Unknown	17	36,926	1	4.2
	16	Unknown	17	12,421	1	12.1
	17	Unknown	17	8180	1	18.7
18	1	Major antigen	74	250,951	3	1.9
	2	Hypothetical protein LOAG_11590	20	11,291	1	21.3
19	1	Calponin homolog OV9M	41	72,439	4	8
	2	Unknown	36	8848	1	19.8
	3	Unknown	34	54,746	1	1.6
	4	Major antigen	33	250,951	1	0.5
	5	Inositol polyphosphate multikinase	29	55,418	1	1.4
	6	Unknown	29	7351	1	10
	7	Unknown	29	12,755	1	6.3
	8	Acetyl-CoA acetyltransferase, mitochondrial	27	72,535	1	1.8
20	1	Collagenase 3	144	31,095	6	21.5
	2	Small heat shock protein ov25-1	54	31,353	1	3.1
	3	unknown	42	30,189	2	11.5
21	1	Collagenase 3	323	31,095	7	28
	2	Hypothetical protein LOAG_06805	40	25,230	1	4.2
	3	Unknown	37	13,565	1	17.4
	4	Unknown	35	54,746	1	1.6
	5	sh2 domain-containing protein 3c	34	86,816	1	1
22	1	Collagenase 3	64	31,095	4	13.8
	2	e3 ubiquitin-protein ligase trim13	29	92,227	2	3.1
	3	Unknown	22	42,073	1	3.4
	4	Isocitrate isopropylmalate dehydrogenase domain-containing protein	17	67,366	1	2.3
	5	PREDICTED: 5-oxoprolinase	17	102,576	2	3.7
	6	putative f-box lrr-repeat protein	17	45,640	1	3.1
	7	N-alpha-acetyltransferase 60	17	19,499	1	7.9
	8	Unknown	17	17,177	1	7.8
	9	Hypothetical protein WUBG_00429	17	55,682	1	2.4
	10	Hypothetical protein DICPUDRAFT_160192	17	7537	1	18.3
	11	Unknown	17	13,878	1	11.1
	12	Cathepsin B	17	73,569	1	2.1
23	1	Unknown	36	22,949	1	5.6
	2	WD-repeat protein 22	33	113,846	1	0.8
	3	sh2 domain-containing protein 3c	29	86,816	2	2.3
	4	Unknown	29	54,746	1	1.6
	5	Glutamate-gated chloride channel subunit beta	29	67,212	1	1.3
	6	Unconventional myosin-IXb	23	231,645	1	0.7
	7	Na-dependent Cl/HCO3 exchanger	17	134,665	1	1.2
24	1	Intermediate filament protein B	95	83,781	4	7
	2	Unknown	32	32,006	1	5.6
	3	Mucin-5AC	19	141,479	1	0.6
	4	Hypothetical protein Y032_0138g2053	19	114,441	1	0.7
	5	Kinesin-like protein KIF3B	19	8000	1	10.3
	6	Cytochrome b5 reductase 4	19	36,260	1	2.6
	7	Histone-lysine N-methyltransferase SETMAR	19	22,618	1	3.8

Potentially immunogenic proteins are known to be found in the 24-kDa region. A total of three, five, and 12 24-kDa proteins were identified in gels 20, 21, and 22, respectively. Interestingly, matrix metalloproteinase-like protein) was located in spots 20, 21, and 22, while cathepsin B was observed in spot 22 only. These two proteins are considered to be antigens in several parasites. Therefore, we predicted their protein properties, including molecular weight, isoelectric point, secretory and transmembrane domains, n-glycosylation, and o-glycosylation (Table 6). The results showed that matrix metalloproteinase-like protein and cathepsin B were secreted proteins through signaling peptides and both of them contained n-glycosylation and o-glycosylation sites. As some clinical manifestations of *G. spinigerum* infection similar to *Angiostrongylus* spp., *Strongyloides* spp., and *Sparganum* spp. infection for example eosinophilic meningitis, larval migration and migration to the brain, distinguishing between *G. spinigerum* infection and the relating parasites might be helpful for diagnosis. Comparison of *G. spinigerum* matrix metalloproteinase-like protein and cathepsin B protein sequences with the highest % homology with protein sequences from humans and from related helminths, including *Angiostrongylus* spp., *Strongyloides* spp., and *Sparganum* spp., was performed. Matrix metalloproteinase-like protein and cathepsin B protein demonstrated 28%–31% and 34%–64% homology to related helminths and humans, respectively. Therefore, these proteins could be potential candidates for novel diagnostic assays to detect gnathostomiasis.

**Table 6:** The protein properties of *G. spinigerum* matrix metalloproteinase-like protein and cathepsin B.

Properties	Software	Matrix metalloproteinase-like protein	Cathepsin B
Molecular weight	Expasy Compute pI/Mw tool	27,661.1 Da	52,709.06 Da
Isolectric point	Expasy Compute pI/Mw tool	7.09	8.54
Signal peptide	SignalP 5.1	Cleavage site between pos. 23 and 24	Cleavage site between pos. 31 and 32
Non-classical secretory	SecretomeP-2.0 Server	Containing signal peptide	Containing signal peptide
Transmembrane protein	TMHMM—2.0	Inside ( pos 1–6), Tmhelix (pos 7–26), outside (27–245)	Outside (1–462)
N-glycosylation	NetNGlyc—1.0	position 41, 59	Position 156, 243
O-glycosylation	NetOGlyc—4.0	position 220, 226, 230, 234, 236	Position 175, 301, 303, 304, 315, 456
% similarity	Clustal omega	28.74% with VDM58434.1 of *Angiostrongylus* spp.	62.21% with VDM57237.1 of *Angiostrongylus*spp.
		30.29% with XP_024501568.1 of *Strongyloides* spp.	64.16% with XP_024507370.1 of Strongyloides spp.
		30.40% with VZI42166.1 of *Sparganum* spp.	34.38% with VZI02086.1 of *Sparganum* spp.
		31.9% with NP_002414.1 of *H. sapiens*	46.91% with NP_071447.1 of *H. sapiens*
Gene ontology	UniProt	Hydrolase, Protease and Metal-binding	Cysteine-type peptidase activity
Molecular weight	Expasy Compute pI/Mw tool	27,661.1 Da	52,709.06 Da
Isolectric point	Expasy Compute pI/Mw tool	7.09	8.54
Signal peptide	SignalP 5.1	Cleavage site between pos. 23 and 24	Cleavage site between pos. 31 and 32
Non-classical secretory	SecretomeP-2.0 Server	Containing signal peptide	Containing signal peptide
Transmembrane protein	TMHMM—2.0	Inside ( pos 1–6), Tmhelix (pos 7–26), outside (27–245)	Outside (1–462)
N-glycosylation	NetNGlyc—1.0	Position 41, 59	Position 156, 243
O-glycosylation	NetOGlyc—4.0	Position 220, 226, 230, 234, 236	Position 175, 301, 303, 304, 315, 456

## Discussion

Our data showed that actin-2 is highly expressed in aL3Gs. In addition, high levels of other *G. spinigerum* structure-related proteins, such as myophilin and actin-1, were also found. Actin is a cytoskeletal protein found in myofibrils in muscle cells and microfilaments in a variety of other cell types^13^. In trematodes, actin has been widely researched. It appears to be a crucial component of the platyhelminth tegument and is engaged in a variety of critical functions, including movements of secretory vesicles, muscle contraction, cytokinesis, and maintenance of cell shape^14^. In parasitic nematodes, cytoskeletal proteins play roles similar to those in trematodes and also have a function in nutrient uptake through transcuticular absorption^15^. The possibility of anthelmintic medications that attack helminth cytoskeletal proteins has piqued researchers' interest. In recent years, research on the microtubular network and its interaction with benzimidazole anthelmintics has been published^16,17^. Therefore, *G. spinigerum* structure-related proteins could be anthelmintic drug targets. In our study, GO classification of all identified aL3Gs proteins highlighted embryo development ending in birth or egg hatching (GO:0009792) as the major protein class expressed in aL3Gs. This protein class controls how an embryo develops over time, from zygote formation to the end of the embryonic life stage^18^. Similarly, transcriptomic analysis of the murine parasite *Heligmosomoides polygyrus* also found this protein class to be the most abundant GO term^19^. In *G. spinigerum*, the life cycle begins when feces containing parasite eggs reach fresh water. Second-stage larvae hatch from the eggs and early third-stage larvae develop after being consumed by cyclopoid copepods. When infected copepods are eaten by various intermediate hosts, such as fish, amphibians and reptiles, the larvae develop into aL3Gs. When the aL3Gs are consumed by definitive hosts, they migrate into the stomach wall and eventually mature into the adult stage, thus completing their life cycle^20^. Because aL3Gs are an intermediate stage of development, proteins related to embryo development ending in birth or egg hatching could contribute to and facilitate the maturation process.

As well as GO classification by biological process terms, various protein classes required for parasite survival and host immunity evasion were also considered. The host immune system's oxidative burst can prevent intracellular parasite invasion and proliferation. Therefore, an antioxidant response promotes parasite survival, reduces inflammation, and changes the metabolism of the host cell^21^. In aL3Gs, the most abundant proteins relating to oxidation–reduction were glutathione S-transferase and peroxiredoxin. Moreover, these two proteins were also identified as *G. spinigerum* antigens (gels 9 and 10 in the 24-kDa region). Helminth glutathione S-transferase represents the main mechanism of detoxifying reactive oxygen intermediates produced by the parasite's endogenous metabolism or by the host immune system^22^. A 28-kDa glutathione-S-transferase was identified as an anti-schistosome vaccine candidate and recently evaluated in human clinical trials^23^. The glutathione-S-transferases have also been identified as potential immunotherapy or chemotherapy targets to treat infection with several other parasites, including *Heligmosomoides polygyrus*, *Onchocerca gutturosa,* and *Dirofilaria immitis*^24^. Peroxiredoxins are cysteine-dependent peroxidases that also play an important role in antioxidant, regulatory, and signaling systems. They are involved in defense against both endogenous and host-derived reactive oxygen species^25^. Treatment of mouse peritoneal macrophages with recombinant *Toxoplasma gondii* peroxiredoxin resulted in the production of IL-12p40 and IL-6^26^. This result suggests that peroxiredoxin induces both humoral and cellular immunological responses. Therefore, it could be used as a toxoplasmosis vaccine antigen. Peroxiredoxins have also been found in several parasitic helminths that affect humans such as *Brugia malayi*^27^, *Onchocerca volvulus*^28^, *Taenia solium*^29^, *Opisthorchis viverrini*^30^, and *Schistosoma japonicum*^31^. Furthermore, these proteins have been reported to be attractive therapeutic targets and vaccine candidates to treat and prevent helminth infections^32^. Nucleoredoxin was one of most abundantly expressed *G. spinigerum* proteins relating to oxidation–reduction. This protein is a newly discovered member of the thioredoxin family and plays a role in cell proliferation and differentiation^33^. Nucleoredoxin also regulates phosphofructokinase activity to balance between glycolysis and the pentose phosphate pathway^34^. However, there is limited information about this protein’s role in parasites. Therefore, the function of nucleoredoxin in *G. spinigerum* needs to be further explored.

Proteases and protease inhibitors are another important protein class expressed in *G. spinigerum*. The most prevalent proteases in aL3Gs were zinc metalloproteinase nas-14, matrix metalloproteinase-like protein, and cathepsin B-like cysteine proteinase 6. These three proteases had limited homology with human proteins. Therefore, they might be potential candidates for anthelmintic drug development. Moreover, they were also identified as *G. spinigerum* antigens (gels 9 and 11 in the 24-kDa region).

The ability of nematodes to molt is critical for their survival and development. Zinc metalloproteases, leucine aminopeptidases, and cysteine proteases are implicated in molting^35,36^. In the parasitic nematode *Haemonchus contortus*, the zinc metalloprotease nas-33 is required for molting and survival^37^. In *Strongyloides ratti*, collagenase plays a role in adult females at the time of migration through the host intestinal mucosa during oviposition^38^. In *Radopholus similis*, an important plant parasitic nematode, the cysteine proteinase cathepsin B-like plays an important role in worm development and hatching^39^.

Owing to the various protease functions in parasitic worms, nematode proteases have been proposed as new anthelmintic and vaccine targets. The most abundant protease inhibitors in aL3Gs were metalloproteinase inhibitor tag-225, serpin I2, and serpin B4. There was no homology between these three protease inhibitors and human proteins. *G. spinigerum* serpins have been identified in excretory–secretory products of aL3Gs and have been shown to react with sera from gnathostomiasis patients^4^. Therefore, they may be good candidates for therapeutic and diagnostic development. Parasite protease inhibitors create a safer environment in the host by suppressing host protease activity and modulating host immunity^40^ . Serpins from the parasitic worms *Brugia malayi*^41^ , *Ancylostoma caninum*^42^, and *Trichuris suis*^43^ have been identified. *Brugia malayi* serpin functions by neutralizing the immunostimulatory properties of the host cathepsin G^44^. *Onchocerca volvulus* serpin reduces the enzymatic activity of a panel of serine proteases including host elastase, chymotrypsin, trypsin, and cathepsin G^45^. Helminth protease inhibitors protect the worms from the hydrolytic action of host proteases and allow them to break through protective barriers and evade immunological responses. Protease inhibitors are, therefore, beneficial to parasite survival and colonization in the host^46^. Consequently, *G. spinigerum* proteases and protease inhibitors might have similar protective roles and may be good anthelmintic candidates.

Several *G. spinigerum* cuticle collagens—cuticle collagen 34, cuticle collagen 36, and collagen alpha-1 (XVII) chain—were identified in aL3Gs. These cuticle collagens had no homology with human proteins. However, these three cuticle collagens were conserved among other nematodes (data not shown). As a result, they may be suitable universal vaccine or drug targets for novel pan-nematicide therapeutics. Moreover, cuticle collagen 14 was identified as a 24-kDa *G. spinigerum* antigen and it showed less homology with protein sequences from other nematodes (data not shown). Therefore, this protein might be a potential target for the development of novel gnathostomiasis diagnostics and vaccines.

The exoskeleton of *Caenorhabditis elegans*, a free-living nematode, comprises a complex collagen matrix. Individual cuticle collagen gene mutations can result in exoskeletal abnormalities that change the shape of a nematode^47^. RNA interference targeted to the cuticle collagen genes of the root-knot nematode *Meloidogyne incognita* caused a 30.80%–35.00% reduction in the number of adult females and a 76.47%–82.59% reduction in the number of eggs. This study demonstrates the role of cuticle collagen genes in the structure and development of the nematode cuticle^48^. Furthermore, external enzymes that induce structural damage to the cuticle, such as papain, bromelain, collagenase, chitinase, and lipase, cause parasitic worms to die. After incubating *Heligmosomoides polygyrus*, *Trichuris muris*, and *Protospirula muricola* with plant cysteine proteases, the nematodes died as a result of cuticle damage^49^. These data suggest that cuticle collagens could be fascinating targets for nematicide development.

Western blot analysis has recently identified diagnostic targets for gnathostomiasis by detecting specific total IgG against a 24-kDa protein in aL3G extract^5^ . This study detected specific IgG subclasses as well as total IgG against a 24-kDa antigen. When compared with other subclasses, IgG4 had the best sensitivity and specificity (91.6% and 87.8%, respectively)^6^. Although the detection of specific IgG against crude worm antigen (CWA) has a high sensitivity and specificity, the antigen preparation procedure is complex, time-consuming and laborious, and batch-to-batch quality is inconsistent. Furthermore, natural sources of *G. spinigerum* are limited and depend on the season and climatic conditions. Therefore, identification of *G. spinigerum* candidate antigens is crucial for recombinant protein production to improve gnathostomiasis diagnosis. The 24-kDa diagnostic protein was first identified as matrix metalloprotease^9^. In another report, matrix metalloproteinase-like protein and cyclophilin with approximate molecular weights of 23–24 kDa were identified as *G. spinigerum* antigens^50^.

However, identification of the protein composition of aL3Gs has been hampered by the lack of genomic sequence information required for proteomic analysis. In our study, the global aL3G antigen profile and specific 24-kDa aL3G antigens were identified using next-generation sequencing transcriptomic information^4^. Matrix metalloproteinase-like protein and cyclophilin were identified in the 24-kDa region, corresponding with previous reports. However, additional *G. spinigerum* antigen candidates were also identified. A total of 74 proteins were observed in 24-kDa bands on our SDS-PAGE gels. Our data suggest that glutathione S-transferase 1, cuticle collagen 14, major antigen, zinc metalloproteinase nas-4, major egg antigen, peroxiredoxin, and superoxide dismutase [Cu–Zn] might also be good candidates to explore for human gnathostomiasis diagnostic assays. On 2-DE, 13 proteins were also observed in 24-kDa bands.

A recombinant *G. spinigerum* matrix metalloproteinase protein has been produced. This protein was shown to have a sensitivity and specificity of 100% in a study comparing 40 patients with 30 healthy controls^51^. In another study, recombinant *G. spinigerum* matrix metalloproteinase protein was analyzed by immunoblot of serum samples from proven and clinically suspected cases of gnathostomiasis, patients with other parasitic diseases, and healthy volunteers. The sensitivity and specificity in this study were 100% and 94.7%, respectively^52^. Although matrix metalloproteinase protein is a potential candidate for *G. spinigerum* diagnostics, it may be important to assess the different isoforms of this protein to improve its diagnostic capability. Our data also show that cathepsin B may be another good diagnostic candidate for human gnathostomiasis.

In this study, SDS-PAGE identified more proteins than 2-DE, possibly because 2-DE cannot resolve proteins that are too basic or too acidic, too large or too small. In addition, highly hydrophobic proteins are hard to dissolve in the 2-DE buffer system^53^. Therefore, integrating SDS-PAGE and 2-DE data from the 24-kDa region might be a useful way to explore novel diagnostic candidates for *G. spinigerum*. This study identified both matrix metalloproteinase like protein and cathepsin B from SDS-PAGE and 2-DE. Furthermore, these proteins were predicted to be secreted proteins and, therefore, might be useful for circulating antigen detection in patient serum. Moreover, both antigens were predicted to have glycosylation. Because glycosylation contributes to protein antigenic properties^54^, this post translational modification might need to be taken into account during any recombinant antigen production. the limitation of proteomics study using other *G. spinigerum* stages is to obtain adequate amount of parasite samples. In conclusion, the data from this proteomic and immunoproteomic analysis could help researchers better understand not only the parasite's biology but also possible targets for future treatments, diagnostics, and vaccines.

## Methods

### Preparation of third-stage *G. spinigerum* larvae

The aL3Gs were obtained from the livers of naturally-infected eels using an acid-pepsin digestion technique. Eel livers were brought from markets in Bangkok. They were chopped and subjected to 1% acid-pepsin at 37 °C for 2 h in a water bath stirred frequently, then were washed with tap water several times. Worms were then collected via a simple sedimentation technique. The collected worms were further identified using a dissecting microscope then washed again with tap water followed by normal saline solution (0.85% NaCl). Specie of *G. spinigerum* was confirmed by morphology. Approximately 20 collected worms were pooled in a microfuge tube and kept in -80 °C prior to analysis.

### Sodium dodecyl sulphate–polyacrylamide gel electrophoresis (SDS-PAGE)

The pooled aL3Gs were ground using a mortar and pestle after short snap freezing. Lysis buffer was then added and further homogenization was performed using an ultrasonicator. After sonication, the mixture was centrifuged at 12 000 rpm for 15 min at 4 °C. The supernatant was collected and the protein concentration was determined by the Bradford method. Proteins from the lysate were separated by 12% SDS-PAGE gel and later stained with Coomassie Brilliant Blue G250 solution (Bio-Rad, Hercules, CA, USA). After running the SDS-PAGE, the gel was cut into 14 rectangles followed by in-gel digestion.

### Two dimensional gel electrophoresis (2-DE)

For the first dimension, a non-linear immobilized pH gradient (IPG) strip (pH 3–10; Amersham Bioscience, USA) was rehydrated overnight in immobilized pH gradient (IPG) sample buffer (8 M urea, 2% (w/v) 3-[(3-cholamidopropyl)dimethlyammonio]-1 propanesulfonate (CHAPS), 15 mM dithiothreitol (DTT), and 0.5% IPG sample buffer) and crude aL3G extract. Isoelectric focusing (IEF) was then performed using the following parameters: 30 V for 14 h; 200 V for 1 h; 500 V for 1 h; 1000 Vfor 1 h; 3500 V for 1 h; and 8000 V for 18 h. After focusing, the strips were equilibrated with DTT for 15 min and with iodoacetamide (C_2_H_4_INO) for 15 min. The strips were then subjected to 12% SDS-PAGE at 120 V until the bromophenol blue dye front reached the bottom of the gel. All three 2-DE gels were stained with silver and the immunoreactive spots in these gels were excised and pooled for mass spectrometry analysis. A different 2-DE gel was used for immunoblotting.

### Immunoblotting

All methods were carried out in accordance with relevant guidelines and regulations. All experimental protocols were approved by the Ethics Committee of the Faculty of Tropical Medicine, Mahidol University (MUTM 2020–058-02). Informed consent was obtained from all subjects and/or their legal guardian(s). Proteins from SDS-PAGE were transferred onto a nitrocellulose membrane. The membrane was cut into five strips and immunoblotting was performed using sera from five different patients as the primary antibody. The proteins separated by 2-DE were transferred onto a nitrocellulose membrane and pooled patient sera was used as the primary antibody. Membranes were blocked using 5% (w/v) non-fat milk in phosphate buffered saline (PBS) for 2 h at room temperature, then rinsed with PBS containing 0.05% (v/v) Tween-20. Serum samples diluted 1:200 in PBS containing 1% non-fat milk were added to the membranes and incubated overnight at 4 °C. After incubation, the membranes were washed three times with PBS containing 0.05% (v/v) Tween-200. Then horseradish peroxidase-conjugated goat anti-human IgG secondary antibodies were added and incubated for 1 h. Immunogen spots were visualized by detection of peroxidase activity using the Ultra TMB-Blotting Solution (ThermoFisher Scientific, UK). Immunoreactive protein spots were excised from silver-stained 2-DE gels and subjected to in-gel digestion.

### In-gel tryptic digestion

Gel slices from SDS-PAGE and gels from 2-DE were destained until colorless. The former was destained with 50% acetonitrile (ACN, Sigma-Aldrich) in 50 mM ammonium bicarbonate (ABC, Sigma-Aldrich), while 30 mM potassium ferricyanide (K_3_Fe(CN)_6_, Sigma-Aldrich) and 100 nM sodium thiosulfate (Na_2_S_2_O_3_, Sigma-Aldrich) was used for the latter. After destaining, gel pieces were incubated in 4 mM DL-Dithiothreitol (DTT, Sigma-Aldrich) at 60 °C for 15 min. The DTT was subsequently removed and proteins were alkylated by adding 250 mM iodoacetamide (ICH_2_CONH_2_, Sigma-Aldrich) and incubated at room temperature in the dark for 30 min. The reaction was quenched with 4 mM DTT and dehydrated in 100% ACN. To digest the proteins, the gel pieces were again rehydrated with 10 ng/µL trypsin in 50 mM ABC and incubated at 37 °C overnight. The peptides were recovered by adding ACN. The supernatant was collected and dried using a vacuum centrifuge (TOMY, Japan). Dried peptides were resuspended in 0.1% formic acid for LC–MS/MS analysis.

### Mass spectrometry

A MicroTOF Q II mass spectrometer interfaced with an Ultimate™ 3000 nano-LC system was used for LC–MS/MS. An Acclaim PepMap RSLC 75 µm × 15 cm nanoviper C18 column with 2 µm particle size and 100 Å pore size (Thermo Scientific, Waltham, MA) was used. Data were acquired using a MicroTOF Q II mass spectrometer set with a scan range of 500–3500 m/z. Mass spectrometry data were analyzed using the MASCOT search engine 2.3 (Matrix Science, Ltd) for peptide identification and a previously reported in-house transcriptomic database^1^ as the reference proteome. Search parameters were set as follows: one miss cleavage; trypsin digestion; 0.8 Da peptide tolerance; ± 0.8 fragment mass tolerance; carbamidomethyl (C) and oxidation (M) variable modifications. Significance threshold was 0.05. The protein abundance was also determined based on the LC-MS/MS output^55^.

### Bioinformatic analysis

After protein identification using the in-house transcriptomic database based on the recently published ES proteome of infective stage *G. spinigerum* larvae^4^, the identified proteins were further classified using the GO database (http://www.geneontology.org) to determine and predict the biological processes affected by these parasitic proteins. Furthermore, the sequences of proteins identified by the transcriptomic data were also subjected to the Basic Local Alignment Search Tool (BLAST) translated (BLAST:blastx) and searched against the human non-redundant protein database to identify possible drug and vaccine targets. The % identity, E-value, and query coverage were reported.

For matrix metalloproteinase-like protein and cathepsin B, property predictions and molecular weight and isoelectric point calculations were performed by the Expasy Compute pI/Mw tool (https://web.expasy.org/compute_pi/). Prediction of signaling peptides, non-classical secretory domains, transmembrane protein domains, n-glycosylation, and o-glycosylation were performed using SignalP version 5.1 (https://services.healthtech.dtu.dk/service.php?SignalP-5.0), SecretomeP version 2.0 Server (https://services.healthtech.dtu.dk/service.php?SecretomeP-2.0), TMHMM version 2.0 (https://services.healthtech.dtu.dk/service.php?TMHMM-2.0), NetNGlyc version 1.0 (https://services.healthtech.dtu.dk/service.php?NetNGlyc-1.0), and NetOGlyc version 4.0 (https://services.healthtech.dtu.dk/service.php?NetOGlyc-4.0), respectively. For sequence alignment, all sequences were retrieved from the non-redundant protein sequence NCBI database. The alignments and identity calculations were performed using the Clustal Omega software.

## Supplementary Information

Supplementary Information 1.

Supplementary Information 2.

## Data Availability

The dataset used in this study might be shared upon reasonable request to Onrapak Reamtong, PhD.
