# Supplementary material for: Protein and antigen profiles of third-stage larvae of Gnathostoma spinigerum assessed with next-generation sequencing transcriptomic information
Source: Sci Rep. 2022 Apr 28;12:6915. doi: 10.1038/s41598-022-10826-4 (PMC9051128; doi:10.1038/s41598-022-10826-4)
Supplement: Supplementary file 2 — Supplementary Information 2. [file 41598_2022_10826_MOESM2_ESM.docx]

Protein and antigen profiles of third-stage larvae of *Gnathostoma spinigerum* assessed with next-generation sequencing transcriptomic information

Kathyleen Nogrado^1#^, Tipparat Thiangtrongjit^1#^, Poom Adisakwattana^2^, Paron Dekumyoy^2^, Sant Muangnoicharoen^3^, Charin Thawornkuno^1^, Onrapak Reamtong^1^*

^1^ Department of Molecular Tropical Medicine and Genetics, Faculty of Tropical Medicine, Mahidol University, Bangkok, 10400, Thailand

^2^ Department of Helminthology, Faculty of Tropical Medicine, Mahidol University, Bangkok, 10400, Thailand

^3^ Department of Clinical Tropical Medicine, Faculty of Tropical Medicine, Mahidol University, Bangkok, 10400, Thailand

^#^Kathyleen Nogrado and Tipparat Thiangtrongjit are co-first authors

*Corresponding author: Onrapak Reamtong, Department of Molecular Tropical Medicine and Genetics, Faculty of Tropical Medicine, Mahidol University, Bangkok, 10400, Thailand

Email: onrapak.rea@mahidol.ac.th

Tel: 66 (0) 2306-9138

Fax: 66 (0) 2306-9139

**Supplementary Figure 1.** Uncropped coomassie blue-stained gel of aL3Gs proteins. The proteins were separated using one-dimensional gel electrophoresis


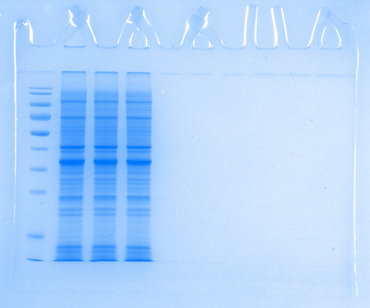


**Supplementary Figure 2.** Uncropped western blot analyses of aL3Gs. Each lane (1-5) is incubated with sera from 5 different individuals diagnosed with gnathostomiasis.


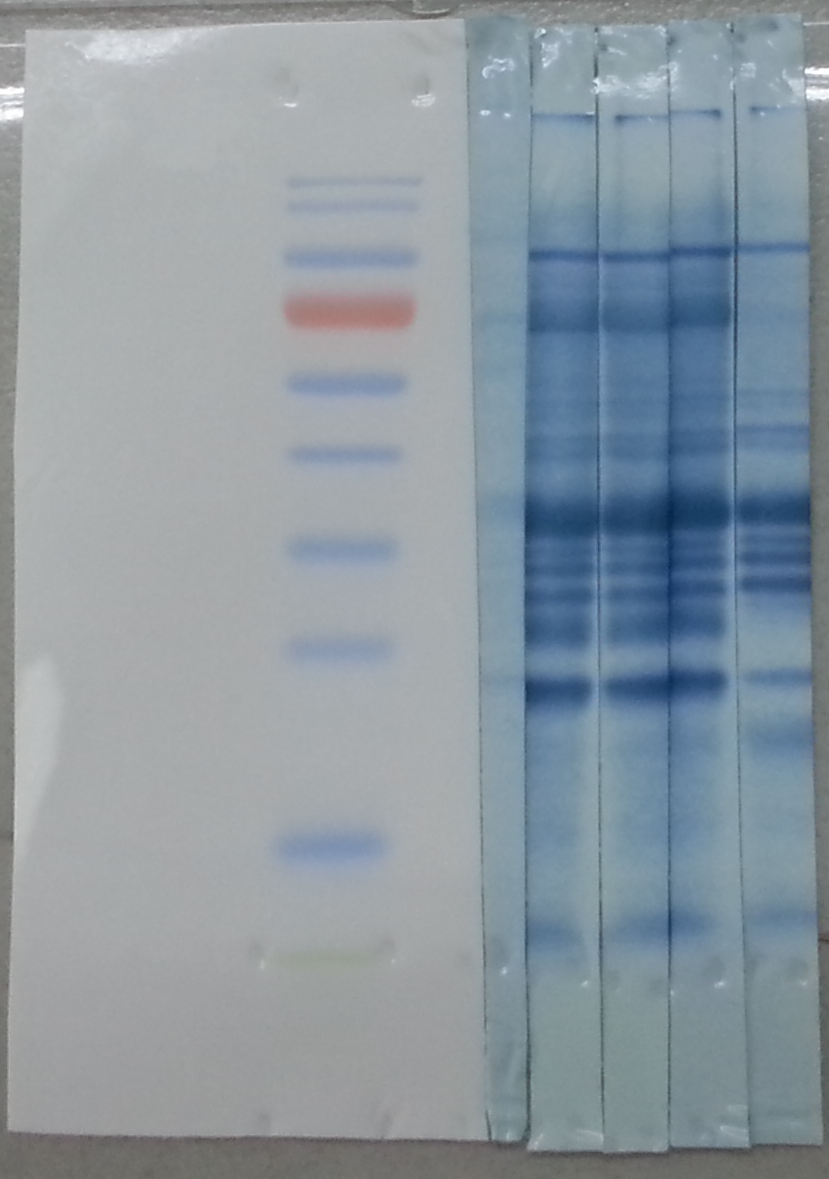


**Supplementary Figure 3.** Uncropped images of protein from crude extract of aL3Gs subjected to two-dimensional electrophoresis (2-DE). Using IEF, the proteins are separated on a linear pH range of 3-10 in the first dimension and followed by 12% SDS-PAGE for the second dimension. The gel on the left is stained with Coomassie blue (A). The right side shows the nitrocellulose membrane with proteins electro-transferred from the gel and then probed with pooled sera from patients diagnosed with gnathostomiasis (B). Numbers at the left of each image indicate protein molecular weight (MW) markers.


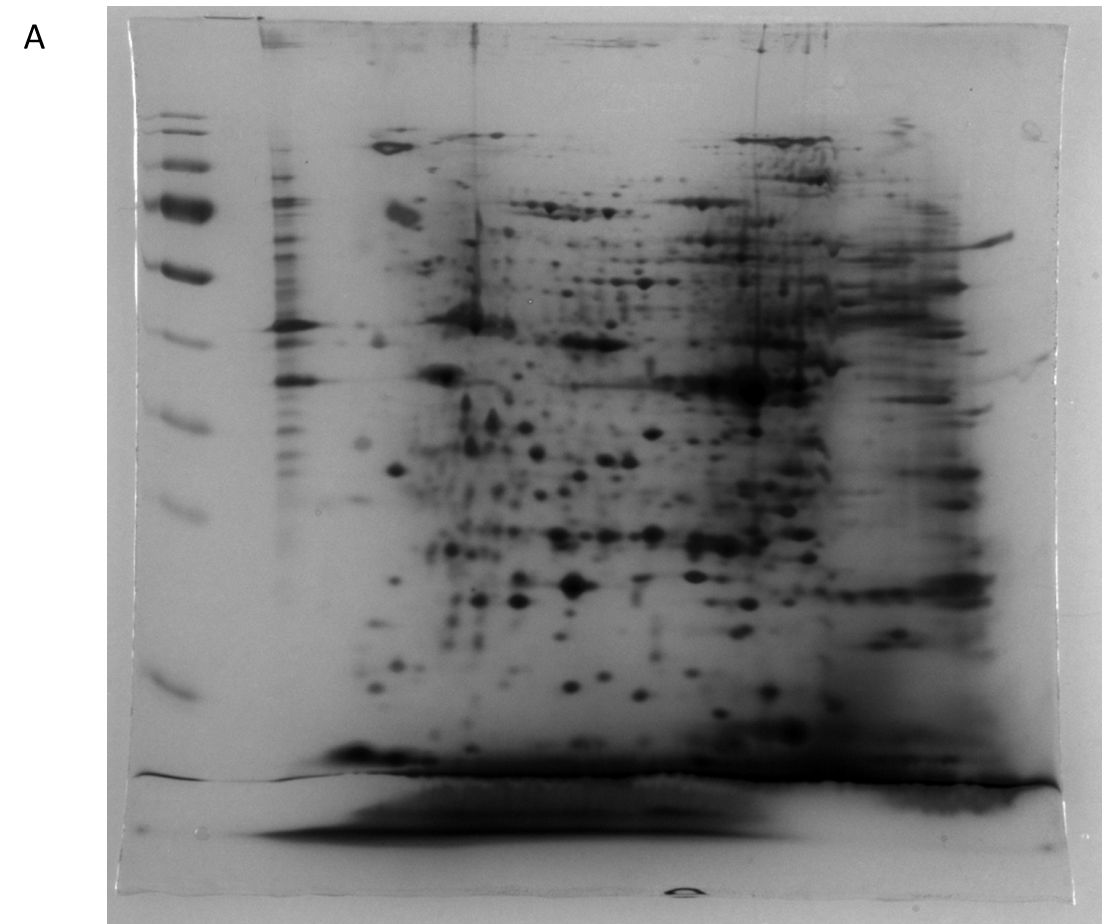


**
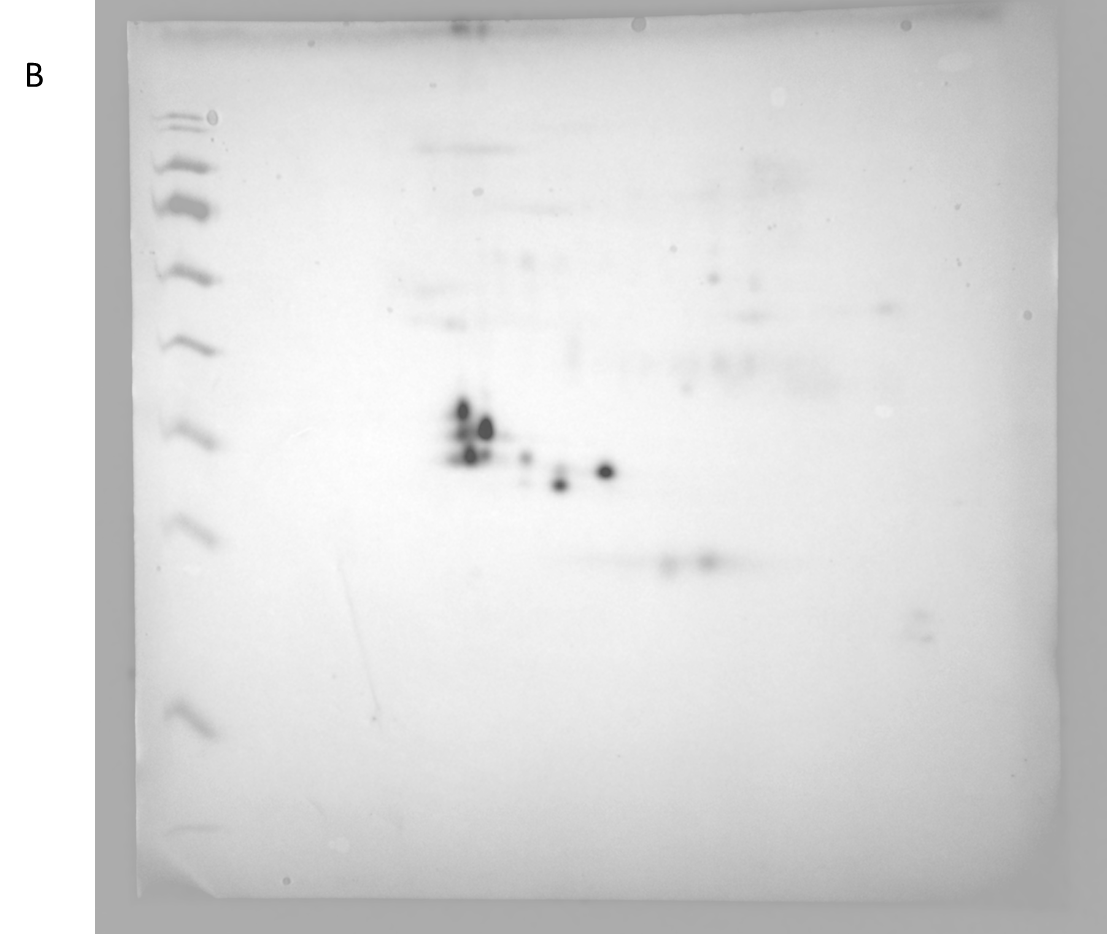
**

**Supplementary Table 1.** Gene ontology of aL3Gs proteome.

| **GO-BiologicalProcess** | **No. of proteins** |
| --- | --- |
| Unknown | 401 |
| GO:0009792//embryo development ending in birth or egg hatching | 33 |
| GO:0008340//determination of adult lifespan | 24 |
| GO:0018991//oviposition | 11 |
| GO:0006096//glycolytic process | 7 |
| GO:0008152//metabolic process | 7 |
| GO:0019915//lipid storage | 7 |
| GO:0055085//transmembrane transport | 6 |
| GO:0000003//reproduction | 5 |
| GO:0010171//body morphogenesis | 5 |
| GO:0040011//locomotion | 5 |
| GO:0040035//hermaphrodite genitalia developmen | 5 |
| GO:0006094//gluconeogenesis | 4 |
| GO:0006457//protein folding | 4 |
| GO:0009987//cellular process;GO:0044238 | 4 |
| GO:0016310//phosphorylation | 4 |
| GO:0030968//endoplasmic reticulum unfolded protein response | 4 |
| GO:0044763;GO:0050794//regulation of cellular process | 4 |
| GO:0048856//anatomical structure development | 4 |
| GO:0071688//striated muscle myosin thick filament assembly | 4 |
| GO:0006508//proteolysis | 3 |
| GO:0040007//growth | 3 |
| GO:0050794//regulation of cellular process | 3 |
| GO:0055114//oxidation-reduction process | 3 |
| GO:0097345//mitochondrial outer membrane permeabilization | 3 |
| GO:1901076//positive regulation of engulfment of apoptotic cell; | 3 |
| GO:0000226//microtubule cytoskeleton organization | 2 |
| GO:0005975//carbohydrate metabolic process | 2 |
| GO:0006099//tricarboxylic acid cycle | 2 |
| GO:0006397//mRNA processing | 2 |
| GO:0006412//translation | 2 |
| GO:0006414//translational elongation | 2 |
| GO:0006520//cellular amino acid metabolic process | 2 |
| GO:0006629//lipid metabolic process | 2 |
| GO:0006810//transport | 2 |
| GO:0006904//vesicle docking involved in exocytosis | 2 |
| GO:0007275//multicellular organismal development | 2 |
| GO:0007411//axon guidance | 2 |
| GO:0007635//chemosensory behavior | 2 |
| GO:0008544//epidermis development | 2 |
| GO:0010466//negative regulation of peptidase activity | 2 |
| GO:0010797//regulation of multivesicular body size involved in endosome transport | 2 |
| GO:0016192//vesicle-mediated transport | 2 |
| GO:0022401//negative adaptation of signaling pathway | 2 |
| GO:0033365//protein localization to organelle | 2 |
| GO:0033396//beta-alanine biosynthetic process via 3-ureidopropionate | 2 |
| GO:0043652//engulfment of apoptotic cell | 2 |
| GO:0045944//positive regulation of transcription from RNA polymerase II promoter | 2 |
| GO:0048477//oogenesis | 2 |
| GO:0005980//glycogen catabolic process | 1 |
| GO:0006071//glycerol metabolic process | 1 |
| GO:0006084//acetyl-CoA metabolic process | 1 |
| GO:0006098//pentose-phosphate shunt | 1 |
| GO:0006164//purine nucleotide biosynthetic process | 1 |
| GO:0006165//nucleoside diphosphate phosphorylation | 1 |
| GO:0006203//dGTP catabolic process | 1 |
| GO:0006281//DNA repair | 1 |
| GO:0006310//DNA recombination | 1 |
| GO:0006486//protein glycosylation | 1 |
| GO:0006487//protein N-linked glycosylation | 1 |
| GO:0006511//ubiquitin-dependent protein catabolic process | 1 |
| GO:0006633//fatty acid biosynthetic process | 1 |
| GO:0006688//glycosphingolipid biosynthetic process | 1 |
| GO:0006892//post-Golgi vesicle-mediated transport | 1 |
| GO:0006897//endocytosis | 1 |
| GO:0006898//receptor-mediated endocytosis | 1 |
| GO:0006950//response to stress | 1 |
| GO:0007016//cytoskeletal anchoring at plasma membrane | 1 |
| GO:0007018//microtubule-based movement | 1 |
| GO:0007126//meiotic nuclear division | 1 |
| GO:0007155//cell adhesion | 1 |
| GO:0007166//cell surface receptor signaling pathway | 1 |
| GO:0007610//behavior | 1 |
| GO:0008292//acetylcholine biosynthetic process | 1 |
| GO:0009236//cobalamin biosynthetic process | 1 |
| GO:0009306//protein secretion | 1 |
| GO:0015991//ATP hydrolysis coupled proton transport | 1 |
| GO:0015992//proton transport | 1 |
| GO:0016482//cytoplasmic transport | 1 |
| GO:0017148//negative regulation of translation | 1 |
| GO:0018105//peptidyl-serine phosphorylation | 1 |
| GO:0018117//protein adenylylation | 1 |
| GO:0018996//molting cycle, collagen and cuticulin-based cuticle | 1 |
| GO:0019509//L-methionine salvage from methylthioadenosine | 1 |
| GO:0022900//electron transport chain | 1 |
| GO:0030042//actin filament depolymerization | 1 |
| GO:0030154//cell differentiation | 1 |
| GO:0030163//protein catabolic process | 1 |
| GO:0030334//regulation of cell migration | 1 |
| GO:0030421//defecation | 1 |
| GO:0030833//regulation of actin filament polymerization | 1 |
| GO:0031987//locomotion involved in locomotory behavior | 1 |
| GO:0032259//methylation | 1 |
| GO:0033275//actin-myosin filament sliding | 1 |
| GO:0034220//ion transmembrane transport | 1 |
| GO:0034551//mitochondrial respiratory chain complex III assembly | 1 |
| GO:0034765//regulation of ion transmembrane transport | 1 |
| GO:0035418//protein localization to synapse | 1 |
| GO:0035626//juvenile hormone mediated signaling pathway | 1 |
| GO:0040018//positive regulation of multicellular organism growth | 1 |
| GO:0040022//feminization of hermaphroditic germ-line | 1 |
| GO:0040032//post-embryonic body morphogenesis | 1 |
| GO:0040034//regulation of development, heterochronic | 1 |
| GO:0042026//protein refolding | 1 |
| GO:0042127//regulation of cell proliferation | 1 |
| GO:0042338//cuticle development involved in collagen and cuticulin-based cuticle molting cycle | 1 |
| GO:0042744//hydrogen peroxide catabolic process | 1 |
| GO:0043087//regulation of GTPase activity | 1 |
| GO:0043401//steroid hormone mediated signaling pathway | 1 |
| GO:0044767;GO:0044707 | 1 |
| GO:0046034//ATP metabolic process | 1 |
| GO:0046474//glycerophospholipid biosynthetic process | 1 |
| GO:0046578//regulation of Ras protein signal transduction | 1 |
| GO:0048609//multicellular organismal reproductive process | 1 |
| GO:0048741//skeletal muscle fiber development | 1 |
| GO:0048873//homeostasis of number of cells within a tissue | 1 |
| GO:0050667//homocysteine metabolic process | 1 |
| GO:0050789//regulation of biological process | 1 |
| GO:0050790//regulation of catalytic activity | 1 |
| GO:0051016//barbed-end actin filament capping; | 1 |
| GO:0051099//positive regulation of binding; | 1 |
| GO:0051131//chaperone-mediated protein complex assembly | 1 |
| GO:0051258//protein polymerization | 1 |
| GO:0070588//calcium ion transmembrane transport | 1 |
